# Supplementary material for: The CCR4–NOT complex maintains liver homeostasis through mRNA deadenylation
Source: Life Sci Alliance. 2020 Apr 1;3(5):e201900494. doi: 10.26508/lsa.201900494 (PMC7119370; doi:10.26508/lsa.201900494)
Supplement: Supplementary file 10 [file LSA-2019-00494_TableS10.docx]

Supplementary Table 10. Sequence lists of primers used in qPCR and polyA tail analyses.

Primer sequences used for Real-time PCR in this study.

| Gene | Forward primer | Reverse primer |
| --- | --- | --- |
| Tnfα | 5’-CGAGTGACAAGCCTGTAGCC-3’ | 5’-TTTGAGATCCATGCCGTTG-3’ |
| Il6 | 5’-CAAAGCCAGAGTCCTTCAGAG-3’ | 5’-GAGCATTGGAAATTGGGGTA-3’ |
| Ccl2 | 5’-AAGAGGATCACCAGCAGCAG-3’ | 5’-TCTGGACCCATTCCTTCTTG-3’ |
| Ccl6 | 5’-TCTTTATCCTTGTGGCTGTCC-3’ | 5’-TGGAGGGTTATAGCGACGAT-3’ |
| Cxcl10 | 5’-GCTGCCGTCATTTTCTGC-3’ | 5’-TCTCACTGGCCCGTCATC-3’ |
| Mx1 | 5’-TTCAAGGATCACTCATACTTCAGC-3’ | 5’-GGGAGGTGAGCTCCTCAGT-3’ |
| Mapk8 | 5’-GGAGGAACGAACTAAGAATGGA-3’ | 5’-CATTGACAGACGGCGAAGA-3’ |
| Tlr3 | 5’-GATACAGGGATTGCACCCATA-3’ | 5’-TCCCCCAAAGGAGTACATTAGA-3’ |
| Ripk3 | 5’-AGGCTTCTAAAGCGAGTGATGT-3’ | 5’-TGAAGTCTTGTCTACCAACTCAGC-3’ |
| Jun | 5’-AGTAGCCCCCAACCTCTTTG-3’ | 5’-GGGACACAGCTTTCACCCTA-3’ |
| Tbp | 5’-GGGGAGCTGTGATGTGAAGT-3’ | 5’-CCAGGAAATAATTCTGGCTCAT-3’ |
| Sp3 | 5’-TGCACCTGTCCCAACTGTAA-3’ | 5’-CCACATCCCGGTATATGACAA-3’ |
| Irf7 | 5’-CTTCAGCACTTTCTTCCGAGA-3’ | 5’-TGTAGTGTGGTGACCCTTGC-3’ |
| Irf9 | 5’-GAGAGGACCCAGTGTTCCTG-3’ | 5’-GGTGAGCAGCAGCGAGTAGT-3’ |
| Trp53 | 5’-ACGCTTCTCCGAAGACTGG-3’ | 5’-AGGGAGCTCGAGGCTGATA-3’ |
| E2f3 | 5’-CAAGGACCCTCCAGCAGAG-3’ | 5’-AGTTCCAGCCTTCGCTTTG-3’ |
| Mapk3 | 5’-AGAGGGCCCCTAACAAGAAC -3’ | 5’- GCAGAGAAGGAGCAGGTAGG-3’ |
| Ccng2 | 5’-GGTTGCTTTGACGGAAGTG -3’ | 5’-AGAAGGTGCACTCCTGATCG-3’ |
| Cdc25a | 5’-TCCCTTTACACACAGGCAGAA-3’ | 5’-GGACTGAAATTTCCTGATTCACTT-3’ |
| Cdt1 | 5’-TGTCAAGGAACAGCACAAGG-3’ | 5’-GCACCTCGTCCACATTGAA-3’ |
| Brca1 | 5’-TTCACCAACATGCCCAAAG-3’ | 5’-AGCTCCTTCACCACGGAAG-3’ |
| Bax | 5’-GTGAGCGGCTGCTTGTCT-3’ | 5’-GGTCCCGAAGTAGGAGAGGA-3’ |
| Bbc3 | 5’-ACCTCAACGCGCAGTACG-3’ | 5’-GAGATTGTACATGACCCTCCAG-3’ |
| Pmaip1 | 5’-CAGATGCCTGGGAAGTCG-3’ | 5’-TGAGCACACTCGTCCTTCAA-3’ |
| Aldh2 | 5’-TGTTCGGGGACGTAAAAGAC-3’ | 5’-TGAGGATTTGCATCACTGGT-3’ |
| Cyp2c44 | 5’-CCAACCCTCGGGATTACAT-3’ | 5’-CAGATTTCAGGTTGTGTTTCTCC-3’ |
| Sod1 | 5’-GCCAATGTGTCCATTGAAGA-3’ | 5’-CACCTTTGCCCAAGTCATCT-3’ |
| Acly | 5’-GAGCCCTCAATGGCATCTT-3’ | 5’-GACGATACAGCCCTTGCTTC-3’ |
| Acox1 | 5’-ACTTGTTTGAGTGGGCCAAG-3’ | 5’-AAACTTCAAAGCTTCGACTGC-3’ |
| Scd1 | 5’-TTCCCTCCTGCAAGCTCTAC-3’ | 5’-CAGAGCGCTGGTCATGTAGT-3’ |
| Gck | 5’-GTGAGGTCGGCATGATTGT-3’ | 5’-TCCACCAGCTCCACATTCT-3’ |
| Cps1 | 5’-CCAGTTTTGCAGTGGAATCA-3’ | 5’-GGTAGCCAATGGTGTCTGCT-3’ |
| Apoa2 | 5’-TTGATGGAGAAGGCCAAGAC-3’ | 5’-CTGACCTGACAAGGGGTGTC-3’ |
| Alb | 5’-TGACCCAGTGTTGTGCAGAG-3’ | 5’-TTCTCCTTCACACCATCAAGC-3’ |
| Rplp0 | 5’-ACTGGTCTAGGACCCGAGAAG-3’ | 5’-TCCCACCTTGTCTCCAGTCT-3’ |
| 18S rRNA | 5’-GCTTAATTTGACTCAACACGGGA-3’ | 5’-AGCTATCAATCTGTCAATCCTGTC-3’ |
| Rplp0 pre-mRNA | 5’-CAGACTGCCACTTGCCAAC-3’ | 5’-GTCCTGGCATTGTCTGTGG-3’ |
| Mx1 pre-mRNA | 5’-TTTCTTTCTACTTTCAACCCCTTG-3’ | 5’-CAGAGGGATCTGTCTCCCAAT-3’ |
| Cxcl10 pre-mRNA | 5’-TCCGGAATCTAAGACCATCAA-3’ | 5’-CCACCATTTCCTGAAAAAGC-3’ |
| Ripk3 pre-mRNA | 5’-CCAGCTCCTGACAACAAGGT-3’ | 5’-GGCTCTCTGGCAGACAAGTT-3’ |
| Pmaip1 pre-mRNA | 5’-TTGAGACTCAGGGTTGTCCA-3’ | 5’-ATCCTCCGGAGTTGAGCAC-3’ |
| Bax pre-mRNA | 5’-GCAGTGGGGACAAGATTCAG-3’ | 5’-GATCAGCTCGGGCACTTTAG-3’ |
| Aldh2 pre-mRNA | 5’-TGCTGAAGCAATCAGTCAGG-3’ | 5’-CAGTCAATTCCGGCTCTTTT-3’ |
| Cps1 pre-mRNA | 5’-CATCCTGCATTCACTTTCATCT-3’ | 5’-CTGCACAGCTTCAGCAAAAA-3’ |
| Gck pre-mRNA | 5’-CCAGGACCCTCAGTGACTTC-3’ | 5’-CCCAGAGTGCTCAGGATGTT-3’ |
| Trp53 pre-mRNA | 5’-ACCTTGTCCAGTGCTTCCAT-3’ | 5’-ATTCAGCTCCCGGAACATCT-3’ |
| Sp3 pre-mRNA | 5’-GACCATATTAATCTGAAAAATTGGAA-3’ | 5’-AAGCGTTTTGAACATTCTGGA-3’ |
| Tbp pre-mRNA | 5’-CCATCTCATTTCTGTTCCATGT-3’ | 5’-GGGGTAGATGTTTTCAAATGC-3’ |
| Irf9 pre-mRNA | 5’-GCGCCTCACTTCCTCTTGTA-3’ | 5’-GCAGCTTTCTCCTCTTCTGG-3’ |
| Cdt1 pre-mRNA | 5’-AGGAATAGAGTCGCGAGCAG-3’ | 5’-CCTCTGCCTACGTCAGCAAT-3’ |
| Cdc25a pre-mRNA | 5’-CCTTGGCTTGGTCTCTCAAA -3’ | 5’-TGACATACAGGCCACGAAGT-3’ |
| Ccl2 pre-mRNA | 5’-TCAGAAGCATCTTTCCTGTCC-3’ | 5’-TCATTGGGATCATCTTGCTG-3’ |

Primer sequences used for Poly(A) tail assay in this study.

| Gene | Forward primer (in the 3’-UTR) |
| --- | --- |
| Cox4i1 | 5’-CTGCGCTCGTTCTGATTTGGGAGAAG-3’ |
| Gapdh | 5’-CAACTTTGTCAAGCTCATTTCCTGGTATG-3’ |
| Pdk4 | 5’-GCATTTGGTTACTTAACCCAAATATCCTGAAC-3’ |
| Ttr | 5’-CACTAAGCATGGTCTGTAGCTATTAAAAGC-3’ |
| Trp53 | 5’-GCCCTCATAGGGTCCATATCCT-3’ |
| Jun | 5’-GCATGTGCTGTGATCATTTA-3’ |
| Cdc25a | 5’-ATGGGTTTGAGTTGCATTTG-3’ |
| Bbc3 | 5’-GCCCAGCCTGTAAGATACTG-3’ |
| Cxcl10 | 5’-ACTCAGAGGAACCTGAAAATG-3’ |
| Cdt1 | 5’-GCCATTGCACAAACTACTCA-3’ |
| Brca1 | 5’-CCTGTTGCTGAGGCATACTA-3’ |
